# Integrative mapping analysis of chicken microchromosome 16 organization

**DOI:** 10.1186/1471-2164-11-616

**Published:** 2010-11-04

**Authors:** Romain Solinhac, Sophie Leroux, Svetlana Galkina, Olympe Chazara, Katia Feve, Florence Vignoles, Mireille Morisson, Svetlana Derjusheva, Bertrand Bed'hom, Alain Vignal, Valérie Fillon, Frédérique Pitel

**Affiliations:** 1 UMR INRA/ENVT Laboratoire de Génétique Cellulaire, INRA, Castanet-Tolosan, 31326, France; 2 Saint-Petersburg State University, Oranienbaumskoie shosse 2, Stary Peterhof, Saint-Petersburg, 198504, Russia; 3 UMR INRA-AgroParisTech Génétique Animale et Biologie Intégrative, INRA, Jouy-en-Josas, 78352, France

## Abstract

**Background:**

The chicken karyotype is composed of 39 chromosome pairs, of which 9 still remain totally absent from the current genome sequence assembly, despite international efforts towards complete coverage. Some others are only very partially sequenced, amongst which microchromosome 16 (GGA16), particularly under-represented, with only 433 kb assembled for a full estimated size of 9 to 11 Mb. Besides the obvious need of full genome coverage with genetic markers for QTL (Quantitative Trait Loci) mapping and major genes identification studies, there is a major interest in the detailed study of this chromosome because it carries the two genetically independent *MHC *complexes *B *and *Y*. In addition, GGA16 carries the ribosomal RNA (*rRNA*) genes cluster, also known as the *NOR *(nucleolus organizer region). The purpose of the present study is to construct and present high resolution integrated maps of GGA16 to refine its organization and improve its coverage with genetic markers.

**Results:**

We developed 79 STS (Sequence Tagged Site) markers to build a physical RH (radiation hybrid) map and 34 genetic markers to extend the genetic map of GGA16. We screened a BAC (Bacterial Artificial Chromosome) library with markers for the *MHC-B*, *MHC-Y *and *rRNA *complexes. Selected clones were used to perform high resolution FISH (Fluorescent *In Situ *Hybridization) mapping on giant meiotic lampbrush chromosomes, allowing meiotic mapping in addition to the confirmation of the order of the three clusters along the chromosome. A region with high recombination rates and containing PO41 repeated elements separates the two *MHC *complexes.

**Conclusions:**

The three complementary mapping strategies used refine greatly our knowledge of chicken microchromosome 16 organisation. The characterisation of the recombination hotspots separating the two *MHC *complexes demonstrates the presence of PO41 repetitive sequences both in tandem and inverted orientation. However, this region still needs to be studied in more detail.

## Background

Sharing the characteristics of a classical avian karyotype, the chicken genome, as standardized by Ladjali-Mohammedi *et al *in 1999 [1], is composed of 9 macrochromosomes including the Z and W sex chromosomes and 30 pairs of microchromosomes. These very small chromosomes range in size between 3.5 and 23 Mb [2,3] and are remarkably gene rich [4]. The draft sequence assembly, comprising the macrochromosomes and the largest microchromosomes, has an overall high quality, apart for numerous gaps and minor local rearrangements [5-7]. However, despite international efforts towards a complete sequence, 9 microchromosomes still remain absent from the current assembly. This can be caused by several factors, such as the difficulty to identify them by cytogenetic approaches or/and problems for cloning and sequencing these (G+C)-rich DNA fragments [4,8]. The contigs which could not be assigned to a chromosome are arranged in a virtual chromosome (Chromosome Unknown, chrUn) and we have previously demonstrated that some of these sequences could be assigned to small microchromosomes [9,10]. Moreover, we have also shown that many chicken cDNA and EST (Expressed Sequence Tag) sequence contigs are absent from the assembly, including in the chrUn fraction, suggesting the total absence of large amounts of the corresponding genomic DNA [10].

GGA16 is the chromosome bearing the *NOR *(Nucleolus Organizing Region, cluster of genes for ribosomal RNA 18S, 5.8S and 28S) and illustrates perfectly the sequencing and mapping problems encountered for avian microchromosomes. Its sequence is far from being complete, with only 433 kb of sequence presented in the current assembly whereas its estimated physical size is between 9 and 11 Mb [11]. Even when taking the *rRNA *genes that cover around 6 Mb and represent 40 to 70% of the chromosome into account [12], a large proportion of GGA16 remains to be sequenced.

Interestingly, the chicken *MHC *(Major Histocompatibility Complex), the role of which is central in the immune response, has also been mapped to GGA16. It is composed of two loci, named the *B *and *Y *complexes [13]. The *B *complex was first identified as a polymorphic blood group system in chicken [14], leading to the first method to identify *MHC *genotypes by serology. Later, the locus was associated with graft rejection [15] and with genetic control of the immune response [16-18], i.e. classical functions of *MHC *locus in vertebrates. The *B *region has been strongly associated with resistance to infectious diseases such as Marek's disease [19].

The first mapping of the *B *complex was conducted by Bloom and Bacon [20] who assigned it to a single microchromosome pair together with the *NOR*. Dominguez-Steglich and her co-authors [21] confirmed this result using non-isotopic *in situ *hybridization. The *Y *complex was discovered later and determined as genetically independent [22,23]. Despite this independence, the two complexes are located together on the same microchromosome, referenced as the pair number 16 [24,25]. The GGA16 genetic linkage group contains only a few microsatellite markers, organised in two independent groups which are separated by 60 cM [26]. Curiously, the recent high-density SNP-based linkage map has no SNP on GGA16 [7]. Finally, no Radiation Hybrid map was constructed for this microchromosome [10]. By comparison with the canonical organization of vertebrate *MHC*, the annotation of the chicken *MHC-B *region shows that only the main small class I and class II core sub-regions are represented in the genome assembly. The extended class I and class III sub-regions are only very partially represented [27,28], and many genes usually present in mammalian *MHC *seem absent. Therefore, despite many attempts at sequencing GGA16 by whole genome or chromosome-specific mapping and sequencing approaches, including very early contig maps built with cosmid clones [29], this chromosome still remains badly covered. Amongst other consequences, this prevents from performing proper Quantitative Trait Loci (QTL) detection in a region which is important for the immune response.

Due to the difficulties encountered in obtaining information on the very specific organization of GGA16 by the sequencing and/or physical BAC chromosome walking strategies, we decided to undertake an integrated approach focussed specifically on this microchromosome. Recently, the order of the *NOR*, *MHC-Y *and *MHC-B *complexes was defined by FISH mapping on mitotic prometaphase and meiotic prophase I pachytene chromosomes [30]. To confirm this organization and build precise genomic markers maps, we integrated three alternative mapping strategies, namely Radiation Hybrid (RH) mapping, genetic mapping, and high resolution FISH mapping on giant lampbrush chromosomes from growing oocytes, allowing the estimation of recombination rates along the chromosome.

## Results

### Development of GGA16 markers

Seventy five RH markers were developed. These were identified as putatively belonging to GGA16 based on several sources of information, including similarity with other *B *and *Y *complex gene sequence and synteny conservation with human. Out of all these potential markers, 13 could finally not be assigned to GGA16 after RH mapping (Additional File 1).

#### - Available GGA16 sequences

A total of 432,983 bp are available for GGA16 in the whole genome assembly [6]. Additional 246,252 bp are designed as "chr16_random", corresponding to contigs assigned to GGA16 without information on their relative positions. Thirty eight primer pairs were designed from these sequences: 30 from GGA16 and 8 from GGA16_random (Additional File 1). In the chicken genome assembly WUGS C2.1/galGal3, the NOR region is located in chicken chromosome 1 [6]; we designed primers in this region to map *NOR *markers, as they are known to be localized on GGA16.

#### - Comparative mapping

Sequence similarities were observed between GGA16 and human chromosome region 6p21 (containing *HLA*, the human *MHC*), or with other human chromosomes (HSA 1, 2, 5, 16, 19, and 21) [6], and therefore chicken chrUn or EST sequence aligning by sequence similarity to these human regions are a potential source of supplementary markers. By this approach, we obtained putative non-*MHC *and non-*NOR *GGA16 markers that should allow linking the three *B*, *Y *and *rRNA *genes loci together. Interestingly, 12 of the chicken EST thus identified presented no similarity with the chicken genome assembly ("no hit" in Additional File 1), suggesting they were left out in the whole genome sequencing process and could thus be part of the missing GGA16 sequences. Eighteen markers were developed and mapped on GGA16 through this strategy (Additional File 1).

#### - BAC screening

Twelve markers, well distributed along the RH map described below, were chosen to screen the Wageningen BAC library [31]. Screening was performed by two-dimensional PCR on DNA pools [31]. Five positive BACs were selected: one for the *NOR *complex (WAG137G04), one for the *Y *complex (WAG523B09), and three for the *B *complex (WAG65G09, WAG258C05 and WAG257D05). The BAC ends were sequenced to develop two supplementary markers (Additional File 1).

The sixth BAC clone (CH261-97F21, from the *B *complex), known to contain a GGA16 fragment [6], was obtained from the BAC PAC Resource Center (BPRC, Oakland, USA).

#### - Identification of the complex of origin for each marker

In a first step, the localization of most of the markers was obtained through previous knowledge - the locations of microsatellites LEI258, MCW312, MCW370 and MCW371 from the *B *complex were known before we started our work [29,32-34] - or *in-silico *sequence analysis: sequences from all the markers developed were compared to the NCBI databases by BLAST (http://blast.ncbi.nlm.nih.gov/Blast.cgi). This allowed assigning 35 fragments to one of the three complexes, thanks to the identification as "18S", "28S", "MHC-B" or "Rfp-Y" fragments (Additional File 1).

Marker 523B09 was assigned to the Y complex because it was developed from a BAC-end sequence obtained from a clone containing marker SEQ113, a fragment from the *Y *complex (see Additional File 1).

In a second step, the assignation of the remaining markers was deduced from their position, relative to known markers, on the RH and/or genetic map.

### Radiation Hybrid map

Altogether, 75 primer pairs enabled successful amplification and the subsequent mapping of the corresponding fragments: 62 mapped to GGA16, 4 to other chromosomes and 9 remained unlinked. The total number of markers assigned to GGA16 rose to 66 with the addition of 4 public SNP RH genotypes obtained from the CNG (Centre National de Génotypages, Evry, France).

**Figure 1:**
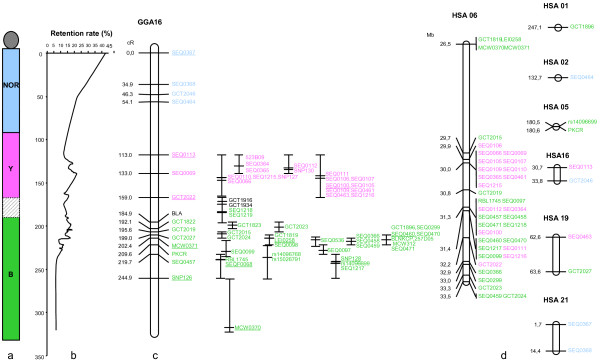
**Radiation hybrid (RH) map of chicken GGA16 and comparison with the human genome**. Marker assignation to one of the three complexes is shown (not at scale) (a). The retention frequency is indicated along the chromosome (b). The framework map is 244.9 cR long (c). Position of markers included in the comprehensive map is indicated with error bars on the right of the framework map. Markers for which the homologous region is approximately localized in the human genome are indicated (d).

After multipoint analysis, the framework map was composed of 15 markers and covers a length of 244.9 cR. The remaining 51 markers were integrated at their best possible locations to build a comprehensive map (Figure 1).

The retention frequency values of the markers along the chromosome were plotted, in order to evaluate the position of the centromere, which usually has a higher retention frequency [35-37].

### Genetic map

**Figure 2:**
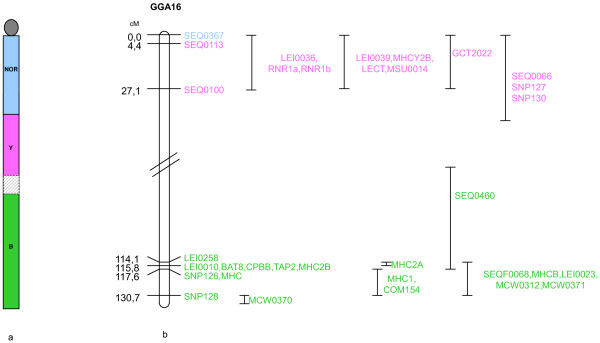
**GGA16 genetic map**. Marker assignation to one of the three complexes is shown (not at scale) (a). The framework map is 130.7 cM long (b). The other markers are indicated with their best location on the right of the framework map.

In order to integrate markers mapped elsewhere, 3 populations were used: our experimental one, the East Lansing backcross one and the Compton backcross one (see Methods section). A total of 16 new informative markers could be added to the GGA16 consensus linkage map [38], eight of which genotyped on our experimental population. Together with the previously available data, the GGA16 genetic map is now composed of 33 markers and is 130.7 cM long (Figure 2).

### Cytogenetic map

#### - FISH on metaphase chromosomes and interphase nuclei

**Figure 3:**
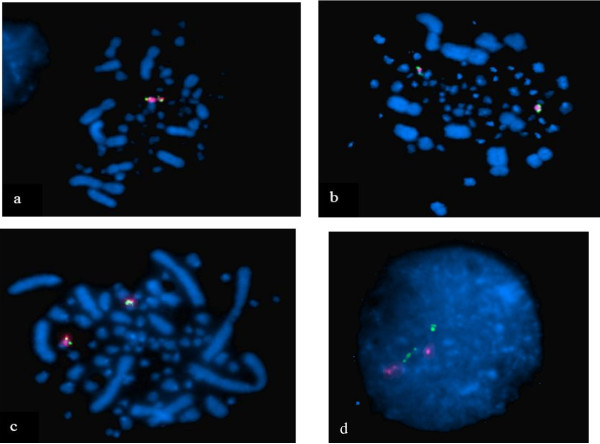
**BAC clones mapped two by two on chicken metaphases**. Three BAC clones were used: WAG137G04 (*NOR*), WAG523B09 (*Y *complex), WAG65G09 (*B *complex). *NOR *(red) and *B *complex (green) are located on the same chromosome (a), as for *Y *complex (green) and *NOR *(red) (b) and for *Y *complex (green) and *B *complex (red) (c). BAC clones WAG523B09 (green, *Y *complex) and WAG65G09 (red, *B *complex) are in the same nuclear area (d). WAG523B09 hybridizes several times the chromatin suggesting the presence of repeat sequences.

Six BAC clones from GGA16 (Table 1) were mapped two by two on chicken metaphase chromosomes and interphase nuclei (Figure 3). These experiments suggest the *B *complex is in q-terminal position, whereas the *NOR *is located at the proximal region of the q arm of GGA16. As already observed, the FISH signals for the *B*, *Y *and *NOR *complexes on condensed mitotic metaphase chromosomes were too close to allow defining their order unequivocally. Intriguingly, WAG523B09, a clone from the Y complex, gave several hybridization signals on interphase nuclei, which might correspond to clusters of repeated sequences dispersed on GGA16 (Figure 3d).

**Table 1:** BAC used for FISH

Complex	Markers	BAC	BAC origin
***B***	SEQ0366	WAG258C05	WAU library

***B***	SEQ0366	WAG257D05	WAU library

***B***	MCW0371	WAG65G09	WAU library

***B***		CH261-97F21	CHORI-261 library, BACPAC Resource Center

***Y***	SEQ0113	WAG523B09	WAU library

**NOR**	SEQ0464	WAG137G04	WAU library

The "Markers" column indicates which marker has been used to screen the Wageningen BAC library (WAU library, Crooijmans et al, 2000).

#### -FISH on lampbrush chromosome 16 (LBC16)

**Figure 4:**
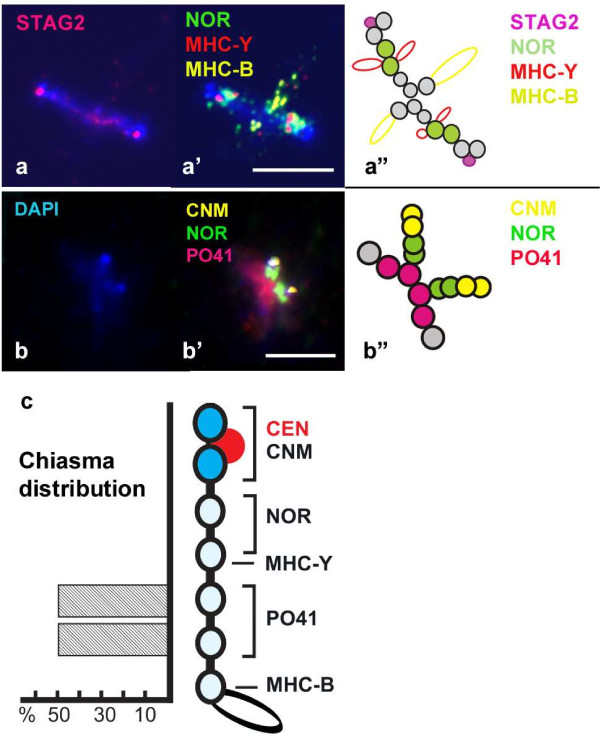
**Chromosome 16 in the lampbrush form**. (a-a'') Centromere localisation (a) by immunostaining for cohesion subunit STAG2 (pink) followed by FISH mapping (a') of BAC clones WAG137G04 (*NOR*, green), WAG523B09 (*MHC-Y *complex, red) and WAG65G09 (*MHC-B *complex, yellow) on the same chromosome. Chromosomes are counterstained with DAPI (blue). (a") The schematic drawing shows the distribution of the signals. Grey circles indicate DAPI-stained chromomeres. (b-b'') High-resolution analysis of distribution of CNM (yellow), PO41 (pink) repeats and BAC clone WAG137G04 (*NOR*, green) (b'). (b) Corresponding DAPI image of the LBC16 is shown. (b") The schematic drawing shows the distribution of the signals. Scale bars = 5 μm. (c) Cytological map of GGA16 in the lampbrush form. Bright DAPI-positive chromomeres are represented by blue circles, the remaining chromomeres by white circles. The red circle indicates centromeric cohesion-enriched structure. The positions of CNM and PO41 repeats as well as *NOR*, *MHC-Y *and *MHC-B *complexes are shown on the right. Chiasma distribution along the bivalent is shown by the histogram on the left.

To define gene order in GGA16 more precisely we hybridized BAC-probes specific for the *B *complex (WAG65G09), the *Y *complex (WAG523B09) and the *NOR *(WAG137G04) to chicken lampbrush chromosomes (LBC) (Figure 4), which allow for higher resolution mapping (e.g. [39]).

GGA16 in the lampbrush form (LBC16) contains 7 chromomeres and has always one chiasma, indicating the position of the meiotic recombination site. Previous studies using immunostaining for cohesin, showed that centromeres on lampbrush microchromosomes, including LBC16, are located at terminal DAPI-positive chromomeres consisting of CNM repeat (chicken nuclear-membrane-associated repeat) [40]. On LBC16 the centromere is situated between two small but prominent terminal chromomeres (Figure 4). The *NOR *complex adjoins these pericentromeric chromomeres and comprises the next two chromomeres. BAC-clones specific for the *Y *complex hybridize to one chromomere next to the *NOR*-positive ones. The *B *complex occupies the distal chromomere (Figure 4).

It is worth noting that FISH with *NOR*-specific probes never produces signals on lateral loops undergoing transcription, but dots in condensed chromomeres. This observation suggests that ribosomal RNA genes are not transcribed at the lampbrush stage, which is consistent with previous data on chicken oocytes (reviewed in [41]). Along with the chromomere occupied by the *Y *complex, the *NOR*-bearing chromomeres form a short "loop-less" region that makes LBC16 easily distinguishable from any other micro-LBCs. In contrast to the *rRNA *genes, at least some of the *MHC *genes seem to be transcribed in LBCs, especially,the *MHC-B *region probes do hybridize to RNA transcripts on lateral loops (Figure 4).

The *B *and *Y *complexes on LBC16 are separated by two chromomeres. These two chromomeres did not hybridize with any of the probes specific to one of the three complexes. Previous detailed analysis of the distribution of CNM and PO41 41 bp repeats using FISH on chicken LBCs [42], showed that the microchromosome we describe here had strong interstitial FISH signals for the PO41 repeat, which are typically subtelomeric. To determine whether the two chromomeres between the *B *and *Y *complexes are composed of repeated sequences, we performed FISH with oligo probes specific to PO41 and CNM repeats followed by FISH with GGA16 specific BAC probes. These experiments clearly showed that the *B *and *Y *complexes are separated by long arrays of PO41 tandem repeat. What is also very special of these PO41-positive chromomeres is that the chiasma observed on LBC16 is invariably associated with one of these two chromomeres, (Figure 4).

## Discussion

Chicken NOR-bearing chromosome GGA16 was originally identified as microchromosome 17 using classical silver staining techniques [43] and re-numbered later as chromosome 16 [25]. Due to technical issues the size of this chromosome was overestimated and its real length is much smaller than chromosome 19 [8]. Past estimations of the total amount of DNA for this chromosome were between 9 and 11 Mb [11]. We have here new estimations for the size of GGA16. The first is deduced from the counting of chromomeres: the average amount of DNA per chromomere in an avian lampbrush microchromosome is between 1.4 and 1.5 Mb [39] and we identified 7 chromomeres in chicken LBC16 (Figure 4), resulting in a size estimation between 9.8 and 10.5 Mb. The second is deduced from our RH map: when mapping the other chicken chromosomes with the chickRH6 panel, we found an average value of 38.7 kb/cR [10], our GGA16 RH map is 244 cR (Figure 1), which suggests a coverage of 9.5 Mb. Therefore, the two size estimations agree together and with the most recently published one [11], and they suggest our RH map covers GGA16 almost entirely. We confirm therefore that approximately 95% of the GGA16 sequence is missing from the current genome assembly.

Three major gene complexes were assigned so far to GGA16: the *NOR*, *MHC-B *and *MHC-Y*. The fact that the *NOR *and *MHC-B *are linked on one microchromosome was discovered 25 years ago [20] and the assignment of *MHC-Y *more recently [24,25].

The chicken *MHC *shows strong similarities with mammalian *MHC *with the presence of genes from class I, II, and III and extended class I regions, but its organization is very different, principally due to the existence of the two genetically independent complexes *MHC-B *and *MHC-Y*. The chicken *MHC-B *complex contains, among other genes, two genes homologous to classical mammalian class I genes: *B-F1 *and *B-F2*, and two genes homologous to classical mammalian class II genes: *B-L1 *and *B-L2 *[13]. It was proposed that the central region of the *B *complex could be considered as minimal and essential, as the limited number of class I and class II genes are found in tight linkage with other genes having functions in the immune response [44]. The *MHC-Y *complex - initially named *Rfp-Y *(restriction fragment pattern Y) - was discovered later, and was defined as a polymorphic locus "*MHC *like" [22]. This second *MHC *region also contains expressed class I and class II genes [45]. The independent genetic assortment of the two complexes has been known for years [22,23], first suggesting they were located on two different chromosomes. However, FISH mapping on chicken chromosomes clearly showed that these two loci both map to GGA16 at a short physical distance, given the small size of this chromosomes [24,25].

For long, the order of the three loci along GGA16 was difficult to resolve as genetic maps were composed of two independent groups, as exemplified by our data (Figure 2) and FISH mapping to mitotic microchromosomes gave signals too close to one another for determining an order (Figure 3). The most commonly admitted model was that the *NOR *region should be situated between the two *MHC *complexes which were situated at the two extremities of the chromosome. In this model, the *NOR *was thought to act as a spacer allowing for meiotic recombination events to take place [25].

Our detailed RH map and high-resolution FISH mapping results on giant lamprush chromosomes from chicken oocytes clearly demonstrate a different order for the three complexe: centromere - *NOR *- *Y *- *B *(Figure 1). The orientation with respect to centromere is obvious both from FISH data in which the centromere is visible and from RH mapping data. For the latter, the position of the centromere on the map is deduced from the retention frequency values along the chromosome, which are usually higher near the centromere [35-37] (Figure 1). The same gene order was recently shown by Delany and her colleagues (2009), based on cytogenetic observations on elongated mitotic and meiotic chromosomes [30]. Thus, with no doubts we can say now that the elusive order of the *NOR*, *Y *and *B *complexes in GGA16 has been established.

The genetic map was enriched by developing polymorphism for markers selected in the RH map and integrating them with markers from the consensus linkage map. The average cR/cM ratio is about 2 when calculated over the whole map length. This relatively low value, as compared to the 4 cR/cM obtained for GGA7 [35] or 6.5 cR/cM for GGA5 [37], is influenced by the disparity in recombination rate observed along the chromosome and is a consequence of the systematic recombination event between the *MHC-Y *and *MHC-B *complexes.

By high resolution mapping on LBC16 we showed that the segment between the *MHC-B *and *MHC-Y *complexes contains many copies of the highly repeated 41 bp tandem repeat, PO41. Until now, PO41 sequences were found to concentrate in the subtelomeric regions of GGA1, GGA2, GGAZ and GGAW [42] and in the current chicken genome assembly, sequences homologous to PO41 are only assigned to the virtual chromosome (chrUn), probably due to difficulties with the repeats in the assembly process. GGA16 is the first identified chicken microchromosome known to contain this repeat. PO41 repeats are (G+C)-rich (63%) and their presence on GGA16 fits the hypothesis of Delany et al.[30] that a GC-rich region separates the *MHC-Y *and *MHC-B*. This organization of the *MHC *complexes seems to be evolutionary conserved in birds, even if not yet demonstrated in Passerines [46]. For instance, a GC-rich satellite MgaSat2 as well as an ultra-long internal telomere array were found between genetically unlinked *MHC-B *and *MHC-Y *loci in turkey [47]. The long arrays of tandem repeats between the two *MHC *complexes in birds can explain their independent genetic segregation. Our data made us return to the idea of highly repeated sequences playing a major role in the formation of recombination hot spots. Indeed, inverted-repeated sequences are known to induce DNA double-strand breaks (DSB) during meiosis in yeast [48], as the first step in a series of events associated with meiotic recombination. Importantly, the same machinery responsible for normal recombination process is involved in palindrome-mediated DSB formation [48]. Although the PO41 repeated units themselves do not tend to form hairpins, they can be found in inverted orientation in the Chicken Genome sequence databases. Moreover, the existence of short tracks of inverted copies was demonstrated for PO41 using sense and antisense oligonucleotide FISH probes applied to chicken LBCs [42]. Thus, the inverted arrays of PO41 repeats can be responsible for DSB generation and consequently a high frequency of recombination events between the *MHC-Y *and *MHC-B *complexes in GGA16. Moreover, the fact that PO41 is intensely transcribed at the position of chiasma formation seems not to be accidental. The relationship between transcription and recombinations hot-spots is well-established (reviewed in [49]).

As it is difficult to obtain the precise distribution and dimension of the PO41 tracks, one can speculate about the degree of complexity of the region between two *MHC *clusters. For instance, part of it could be homologous to a recently cloned sequence from the zebrafinch germ-line restricted chromosome (GRC sequence) [50].

It is not excluded that the region of interest can also be interspersed with coding sequences which remain unsequenced due to compositional reasons. If so, the 12 EST found by examining sequences similar to GGA16 in the human genome and having no similarity with the chicken genome assembly (Additional File 1) could illustrate this case: they all map to the region between the two complexes, or near this portion of the map (Figure 1).

Comparative mapping with the human genome using the EST data reveals that in addition to the known and obvious HSA 6p21 region containing the human MHC (HLA), several other human chromosomes show limited homology or synteny conservation with GGA16 (Figure 1). The HSA01 terminal region and the HSA16 short arm region, which were already suspected to have a region of conserved synteny with GGA16 are confirmed here, both for a short genomic fragment. Although our data confirm the assignment of these regions to GGA16, the issue as to whether these fragments showing sequence similarities really correspond to evolutionary orthologous fragments remains unresolved. A larger conservation of synteny fragments is confirmed with HSA05 and a new human chromosome is shown to share synteny fragments with GGA16: HSA19, the most gene-rich human chromosome. The region of sequence similarity stands at the end of the HSA19 q arm, known to be nearly absent from the actual chicken sequence and genetic map [7]. This human region was already known to share synteny conservation with microchromosomes 30 and 32 [10] and chromosome 11 [51].

The GGA16 sequence coverage is still far from being complete but our addition of EST improves the knowledge on this microchromosome. One could have expected a better sequence coverage with the release of a novel Gallus gallus 12x 454 sequence assembly (http://genome.wustl.edu/genomes/view/gallus_gallus/). But a BLAST analysis of these new contigs with the available sequence assembly reveals only a partial coverage of the GGA16 sequences in this lastest sequence data source (Additional File 2).

## Conclusions

Our data confirm and refine the GGA16 chromosome organization, by using three integrated mapping strategies: RH mapping, genetic mapping and high resolution FISH, especially on lampbrush chromosomes from growing oocytes which were shown here to provide a unique opportunity to study recombination hot spots and to analyse their molecular context. The markers developed here may be useful to improve the GGA16 sequence assembly, and for QTL mapping studies, for which this chromosome is usually missing. The high-recombining region bearing a systematic chiasma separating the two *MHC *complexes is open for further detailed research.

## Methods

Several sources of DNA sequence fragments putatively belonging to GGA16 were used: chicken EST, chicken chrUn sequence and chicken BAC clones. Primer data and other information on the sequences used for marker development are given in Additional File 1. Primers for RH mapping or BAC screening were chosen using the Primer3 server (http://frodo.wi.mit.edu/primer3/).

### Comparative mapping

Human genes from regions for which available comparative mapping data suggested conservation of synteny with GGA16 were selected for marker development. Chicken EST sequence alignments to these genes were visualised by using the ICCARE software [52], to guide the primers design as described in [37].

Additional chicken chrUn contigs showing alignment with the same human regions were selected from the Chicken (*Gallus gallus*) Genome Browser Gateway at UCSC [6], using the May 2006 chicken genome assembly.

Precise positions on the human genome of the chicken RH markers developed were deduced from a BLAST analysis of their sequences - or the sequence of their chicken contig - to the human Mar. 2006 (hg18) assembly [6].

Alignment of the 454 contigs (http://genome.wustl.edu/genomes/view/gallus_gallus/) to the chicken May 2006 (galGal3) assembly (UCSC) was done by BLAST analysis.

### BAC screening

BAC clones from the genomic region of interest were obtained by screening the Wageningen BAC library [31] with markers selected from the GGA16 RH map. PCR amplifications were carried out as for RH mapping (see below), first in BAC superpools, then in pools from the positive superpools. PCR products were analysed on 2% agarose gels, electrophoresed in 1X TBE buffer, and visualized by ethidium bromide staining. DNA was extracted from BAC clones by alkaline lysis according to Qiagen procedures (midi kit, Qiagen).

### BAC end sequencing

Sequence reactions were performed for both ends of BAC clones using the diChloroRhodamine Prism AmpliTaq FS Big Dye Terminator V3.1 kit (Applied Biosystems) and M13 forward or M13 reverse primers. Sequences were analysed on an ABI 3730 sequencer (Applied Biosystems). Sequences providing new information were selected for the development of additional RH markers.

### Radiation hybrid mapping

#### PCR amplification

PCR amplifications were carried out for each marker in 15 μl reactions containing 25 ng DNA from the chickRH6 panel [53], 0.4 μM of each primer, 0.25 units Taq polymerase (GoTaq, Promega), 1.5 or 2 mM MgCl2, 0.2 mM dNTP on a GeneAmp PCR System 9700 thermocycler (Applied Biosystems). The first 5 min denaturation step was followed by 32 to 40 cycles, each consisting of denaturation at 94°C for 30 s, annealing at Tm for 30 s and elongation at 72°C for 30 s. PCR products were analysed on 2% agarose gels, electrophoresed in 1X TBE buffer, and visualized by staining with ethidium bromide.

Each marker was genotyped twice and a third genotyping was performed in case of discrepancy between the first two experiments.

#### RH map construction

The genotyping data obtained were analysed with the Carthagene software [54]. A group of markers for GGA16 was defined by two-point analysis using a LOD threshold of 8. By using all the markers from this group, a 1000:1 framework map (a map whose likelihood is at least 1000-fold higher than the next possible highest likelihood using the same markers in alternate orders) was built under a haploid model. This framework was constructed using a stepwise locus-adding strategy, starting from the triplet of markers whose order is the most likely ("buildfw" option). The different provisional framework maps were checked by using a simulated annealing greedy algorithm, testing for possible improvements of the map by inversion of large fragments, and a flips algorithm testing all possible local permutations within a sliding window of six markers. After validation of the framework map built under the haploid model, the distances between the framework markers were re-evaluated under a diploid model. Finally, markers not included in the framework map were mapped relative to it, to determine their most likely positions.

All maps were drawn with MapChart 2.0 [55].

### Genetic mapping

PCR amplifications were performed as for RH mapping, except for the occasional use of fluorescent primers. Genotypings were performed through different methods, according to the marker (Additional File 1).

Most markers were genotyped by SSCP (Single-Strand Conformation Polymorphism) and silver staining [56,57]. The microsatellite markers were analysed on ABI sequencers (ABI 3700 and ABI 3100, Applied Biosystems). Two markers were genotyped by PCR-RFLP (Restriction Fragment Length Polymorphism). Three markers were analysed by HRM (High Resolution Melting) on a LightCycler480 (Roche): after PCR on a thermocycler 9700 (Applied Biosystem), a dissociation curve was realised by adding LCGreen dye (Bioke): 95°C for 10 minutes, cooling to 40°C for 1 second (ramp rate 2.2°C/s), raising the temperature to 45°C (1s, ramp rate 4.4°C/s) and then raising the temperature to 95°C with 25 fluorescent acquisitions per degree Celsius. The genotypes were analysed with the LightCycler 480 software release 1.5.0.

Segregation analyses were performed in the international East Lansing and Compton chicken reference back-cross mapping populations [58,59]. An experimental population derived from a Fayoumi ancestor, consisting of two chicken half-sib families (Sire1 × 5 females, 115 offspring; Sire2 × 6 females, 94 offspring) was also used to build the genetic map. Linkage analysis was performed using CriMap version 2.4 software [60]. The "build" option was used to order markers within the linkage group, with the same 1000:1 stringency as for the RH map (PUK_LIKE_TOL set to 3.0). The "flips" option was used to examine the order of the different loci by inverting every two or three loci.

### Fluorescence *in situ *hybridisation on metaphase spreads

FISH was carried out on metaphase spreads obtained from embryo fibroblast cultures arrested with 0.06 μg/ml colcemid and fixed by standard procedures. Two-colour FISH was performed by labelling 100 ng of each selected BAC with digoxigenin-11-dUTP (Roche Diagnostics) or biotin-16-dUTP (Roche Diagnostics) by random priming. Probes labelled with biotin and with digoxigenin were precipitated together with ethanol and hybridised on the metaphases for 24 hours at 37°C in a Dakomation hybridizer after denaturation at 72°C for 8 min. Biotin-labelled probes were detected with Alexa 594 antibodies (Invitrogen) and digoxigenin-labelled ones with Alexa 488 antibodies (Invitrogen). Chromosomes were counterstained with DAPI (4', 6-diamidino-2-phenilindole-dihydrochloride) in Vectashield antifade solution (Clinisciences). Two-colour FISH was performed either with one probe labelled in each colour or with groups of two or three clones for each colour. The hybridised metaphases were screened with a Zeiss fluorescence microscope and a minimum of twenty spreads was analysed for each experiment. Spot-bearing metaphases were captured and analysed with a cooled CCD camera using Cytovision software (Applied Imaging).

### Fluorescence *in situ *hybridisation on LBC

Chicken LBC were isolated manually from oocytes of 1.0-1.5 mm diameter according to standard techniques (http://www.exeter.ac.uk/projects/lampbrush/). After overnight fixation in 70% ethanol, preparations were dehydrated in a 70-80-96% ethanol series and air-dried. FISH with BAC clones on LBCs was performed as described [39]. Oligonucleotide probes specific for the 41-bp tandem repeats CNM and PO41 [42] were also used for DNA/(DNA+RNA) hybridization on LBC16.

Before FISH some preparations were stained with an antibody against STAG2, one of cohesin subunits, as previously described [40] to map the centromere on LBC16. After immunostaining and image acquisition, slides were washed in 2xSSC at 42°C, dehydrated in ethanol and air dried before applying FISH probes. LBC16 identified by FISH were used to study chiasma distribution within bivalents. Chiasmata were defined as criss-crosses or tight conjunctions sites of homologous chromosomes.

## Authors' contributions

SL, RS, KF, FV, MM, OC, BB and FP carried out the molecular studies. RS, SG, SD, and VF completed the cytogenetic experiments. MM made the RH panel and the very first RH map of GGA16. SL and FP built the final maps. FP and VF conceived the study, and participated in its design and coordination. FP drafted the manuscript. SG, BB, AV, SL and VF finalized the manuscript. All authors read and approved the manuscript.

## Supplementary Material

Additional File 1 **Markers used in this study**. File format: Excel Click here for file

Additional File 2 **454 contigs distribution on the chicken sequence assembly**. The 454 contigs (http://genome.wustl.edu/genomes/view/gallus_gallus/) were aligned to the available chicken sequence assembly (galGal 3, http://genome.ucsc.edu/index.html). The 7 matching contigs are represented along the GGA16 sequence. File format: PowerPoint Click here for file

## References

[B1] Ladjali-Mohammedi K Bitgood JJ Tixier-Boichard M Ponce De Leon FA International system for standardized avian karyotypes (ISSAK): standardized banded karyotypes of the domestic fowl (Gallus domesticus) Cytogenet Cell Genet 1999 86 271 276 10.1159/000015318 10575225

[B2] Bloom SE Delany ME Muscarella DE RJ Etches AG. Guelph Constant and variable features of the avian chromosomes Manipulation of the Avian Genome 1993 Canada: CRC press 39 59

[B3] Pichugin AM Galkina SA Potekhin AA Punina EO Rautian MS Rodionov AV [Determination of the minimum size of Gallus gallus domesticus chicken microchromosome by a pulse electrophoresis method] Genetika 2001 37 657 660 11436558

[B4] ICGSC, Consortium) ICGS Sequence and comparative analysis of the chicken genome provide unique perspectives on vertebrate evolution Nature 2004 432 695 716 10.1038/nature03154 15592404

[B5] Ensembl Genome Browser http://www.ensembl.org/index.html

[B6] UCSC, Genome, Bioinformatics, Site http://genome.ucsc.edu/index.html

[B7] Groenen MA Wahlberg P Foglio M Cheng HH Megens HJ Crooijmans RP Besnier F Lathrop M Muir WM Wong GK Gut I Andersson L A high-density SNP-based linkage map of the chicken genome reveals sequence features correlated with recombination rate Genome Res 2009 19 510 519 10.1101/gr.086538.108 19088305 PMC2661806

[B8] Masabanda JS Burt DW O'Brien PC Vignal A Fillon V Walsh PS Cox H Tempest HG Smith J Habermann F Schmid M Matsuda Y Ferguson-Smith MA Crooijmans RP Groenen MA Griffin DK Molecular cytogenetic definition of the chicken genome: the first complete avian karyotype Genetics 2004 166 1367 1373 10.1534/genetics.166.3.1367 15082555 PMC1470793

[B9] Douaud M Feve K Gérus M Fillon V Bardes S Gourichon D Dawson DA Hanotte O Burke T Vignoles F Morisson M Tixier-Boichard M Vignal A Pitel F Addition of the microchromosome GGA25 to the chicken genome sequence assembly through radiation hybrid and genetic mapping BMC Genomics 2008 9 129 10.1186/1471-2164-9-129 18366813 PMC2275740

[B10] Morisson M Denis M Milan D Klopp C Leroux S Bardes S Pitel F Vignoles F Gérus M Fillon V Douaud M Vignal A The chicken RH map: current state of progress and microchromosome mapping Cytogenet Genome Res 2007 117 14 21 10.1159/000103160 17675840

[B11] Delany ME Gessaro TM Rodrigue KL Daniels LM Chromosomal mapping of chicken mega-telomere arrays to GGA9, 16, 28 and W using a cytogenomic approach Cytogenet Genome Res 2007 117 54 63 10.1159/000103165 17675845

[B12] Delany ME Krupkin AB Molecular characterization of ribosomal gene variation within and among NORs segregating in specialized populations of chicken Genome 1999 42 60 71 10.1139/gen-42-1-60 10208002

[B13] Miller MM Bacon LD Hala K Hunt HD Ewald SJ Kaufman J Zoorob R Briles WE 2004 Nomenclature for the chicken major histocompatibility (B and Y) complex Immunogenetics 2004 56 261 279 15257423 10.1007/s00251-004-0682-1

[B14] Briles WE McGibbon WH Irwin MR On multiple alleles effecting cellular antigens in the chicken Genetics 1950 35 633 652 14793708 10.1093/genetics/35.6.633 PMC1224328

[B15] Schierman LW Nordskog AW Relationship of blood type to histocompatibility in chickens Science 1961 134 1008 1009 10.1126/science.134.3484.1008 13747603

[B16] Karakoz I Krejcí J Hála K Blaszczyk B Hraba T Pekárek J Genetic determination of tuberculin hypersensitivity in chicken inbred lines Eur J Immunol 1974 4 545 548 10.1002/eji.1830040805 4213124

[B17] Miggiano VC Birgen I Pink JR The mixed leukocyte reaction in chickens. Evidence for control by the major histocompatibility complex Eur J Immunol 1974 4 397 401 10.1002/eji.1830040602 4278328

[B18] Günther E Balcarová J Hála K Rüde E Hraba T Evidence for an association between immune responsiveness of chicken to (T, G)-A--L and the major histocompatibility system Eur J Immunol 1974 4 548 553 10.1002/eji.1830040806 4413330

[B19] Briles WE Stone HA Cole RK Marek's disease: effects of B histocompatibility alloalleles in resistant and susceptible chicken lines Science 1977 195 193 195 10.1126/science.831269 831269

[B20] Bloom SE Bacon LD Linkage of the major histocompatibility (B) complex and the nucleolar organizer in the chicken. Assignment to a microchromosome J Hered 1985 76 146 154 3998437

[B21] Dominguez-Steglich M Auffray C Schmid M Linkage of the chicken MHC to the nucleolus organizer region visualized using non-isotopic in situ hybridization J Hered 1991 82 503 505 1795102 10.1093/oxfordjournals.jhered.a111138

[B22] Briles WE Goto RM Auffray C Miller MM A polymorphic system related to but genetically independent of the chicken major histocompatibility complex Immunogenetics 1993 37 408 414 10.1007/BF00222464 8436415

[B23] Miller MM Goto R Bernot A Zoorob R Auffray C Bumstead N Briles WE Two Mhc class I and two Mhc class II genes map to the chicken Rfp-Y system outside the B complex Proc Natl Acad Sci USA 1994 91 4397 4401 10.1073/pnas.91.10.4397 7910407 PMC43792

[B24] Fillon V Zoorob R Yerle M Auffray C Vignal A Mapping of the genetically independent chicken major histocompatibility complexes B@ and RFP-Y@ to the same microchromosome by two-color fluorescent in situ hybridization Cytogenet Cell Genet 1996 75 7 9 10.1159/000134445 8995478

[B25] Miller MM Goto RM Taylor RLJ Zoorob R Auffray C Briles RW Briles WE Bloom SE Assignment of Rfp-Y to the chicken major histocompatibility complex/NOR microchromosome and evidence for high-frequency recombination associated with the nucleolar organizer region Proc Natl Acad Sci USA 1996 93 3958 3962 10.1073/pnas.93.9.3958 8632997 PMC39467

[B26] Groenen MA Cheng HH Bumstead N Benkel BF Briles WE Burke T Burt DW Crittenden LB Dodgson J Hillel J Lamont S de Leon AP Soller M Takahashi H Vignal A A consensus linkage map of the chicken genome Genome Res 2000 10 137 147 10645958 10.1101/gr.10.1.137 PMC310508

[B27] Kaufman J Milne S Göbel TW Walker BA Jacob JP Auffray C Zoorob R Beck S The chicken B locus is a minimal essential major histocompatibility complex Nature 1999 401 923 925 10.1038/44856 10553909

[B28] Shiina T Briles WE Goto RM Hosomichi K Yanagiya K Shimizu S Inoko H Miller MM Extended gene map reveals tripartite motif, C-type lectin, and Ig superfamily type genes within a subregion of the chicken MHC-B affecting infectious disease J Immunol 2007 178 7162 7172 17513765 10.4049/jimmunol.178.11.7162

[B29] Guillemot F Billault A Pourquié O Béhar G Chaussé AM Zoorob R Kreibich G Auffray C A molecular map of the chicken major histocompatibility complex: the class II beta genes are closely linked to the class I genes and the nucleolar organizer EMBO J 1988 7 2775 2785 3141149 10.1002/j.1460-2075.1988.tb03132.x PMC457068

[B30] Delany ME Robinson CM Goto RM Miller MM Architecture and organization of chicken microchromosome 16: order of the NOR, MHC-Y, and MHC-B subregions J Hered 2009 100 507 514 10.1093/jhered/esp044 19617522

[B31] Crooijmans RP Vrebalov J Dijkhof RJ van der Poel JJ Groenen MA Two-dimensional screening of the Wageningen chicken BAC library Mamm Genome 2000 11 360 363 10.1007/s003350010068 10790534

[B32] Crooijmans RP Dijkhof RJ van der Poel JJ Groenen MA New microsatellite markers in chicken optimized for automated fluorescent genotyping Animal Genetics 1997 28 427 437 10.1111/j.1365-2052.1997.00205.x 9589584

[B33] McConnell SK Dawson DA Wardle A Burke T The isolation and mapping of 19 tetranucleotide microsatellite markers in the chicken Anim Genet 1999 30 183 189 10.1046/j.1365-2052.1999.00454.x 10442979

[B34] Buitenhuis AJ Rodenburg TB Siwek M Cornelissen SJ Nieuwland MG Crooijmans RP Groenen MA Koene P Bovenhuis H van der Poel JJ Identification of QTLs involved in open-field behavior in young and adult laying hens Behav Genet 2004 34 325 333 10.1023/B:BEGE.0000017876.82142.73 14990871

[B35] Morisson M Jiguet-Jiglaire C Leroux S Faraut T Bardes S Feve K Genet C Pitel F Milan D Vignal A Development of a gene-based radiation hybrid map of chicken Chromosome 7 and comparison to human and mouse Mamm Genome 2004 15 732 739 10.1007/s00335-004-3003-y 15389321

[B36] Rabie TS Crooijmans RP Morisson M Andryszkiewicz J van der Poel JJ Vignal A Groenen MA A radiation hybrid map of chicken Chromosome 4 Mamm Genome 2004 15 560 569 10.1007/s00335-004-2362-8 15366376

[B37] Pitel F Abasht B Morisson M Crooijmans RP Vignoles F Leroux S Feve K Bardes S Milan D Lagarrigue S Groenen MA Douaire M Vignal A A high-resolution radiation hybrid map of chicken chromosome 5 and comparison with human chromosomes BMC Genomics 2004 5 66 10.1186/1471-2164-5-66 15369602 PMC521070

[B38] Schmid M Nanda I Guttenbach M Steinlein C Hoehn M Schartl M Haaf T Weigend S Fries R Buerstedde JM Wimmers K Burt DW Smith J A'Hara S Law A Griffin DK Bumstead N Kaufman J Thomson PA Burke T Groenen MA Crooijmans RP Vignal A Fillon V Morisson M Pitel F Tixier-Boichard M Ladjali-Mohammedi K Hillel J Mäki-Tanila A Cheng HH Delany ME Burnside J Mizuno S First report on chicken genes and chromosomes 2000 Cytogenet Cell Genet 2000 90 169 218 10.1159/000056772 11124517

[B39] Galkina S Deryusheva S Fillon V Vignal A Crooijmans R Groenen M Rodionov A Gaginskaya E FISH on avian lampbrush chromosomes produces higher resolution gene mapping Genetica 2006 128 241 251 10.1007/s10709-005-5776-7 17028954

[B40] Krasikova A Deryusheva S Galkina S Kurganova A Evteev A Gaginskaya E On the positions of centromeres in chicken lampbrush chromosomes Chromosome Res 2006 14 777 789 10.1007/s10577-006-1085-y 17115332

[B41] Gaginskaya E Kulikova T Krasikova A Avian lampbrush chromosomes: a powerful tool for exploration of genome expression Cytogenet Genome Res 2009 124 251 267 10.1159/000218130 19556778

[B42] Deryusheva S Krasikova A Kulikova T Gaginskaya E Tandem 41-bp repeats in chicken and Japanese quail genomes: FISH mapping and transcription analysis on lampbrush chromosomes Chromosoma 2007 116 519 530 10.1007/s00412-007-0117-5 17619894

[B43] Auer H Mayr B Lambrou M Schleger W An extended chicken karyotype, including the NOR chromosome Cytogenet Cell Genet 1987 45 218 221 10.1159/000132457 3691189

[B44] Kaufman J Jacob J Shaw I Walker B Milne S Beck S Salomonsen J Gene organisation determines evolution of function in the chicken MHC Immunol Rev 1999 167 101 117 10.1111/j.1600-065X.1999.tb01385.x 10319254

[B45] Afanassieff M Goto RM Ha J Sherman MA Zhong L Auffray C Coudert F Zoorob R Miller MM At least one class I gene in restriction fragment pattern-Y (Rfp-Y), the second MHC gene cluster in the chicken, is transcribed, polymorphic, and shows divergent specialization in antigen binding region J Immunol 2001 166 3324 3333 11207288 10.4049/jimmunol.166.5.3324

[B46] Balakrishnan CN Ekblom R Völker M Westerdahl H Godinez R Kotkiewicz H Burt DW Graves T Griffin DK Warren WC Edwards SV Gene duplication and fragmentation in the zebra finch major histocompatibility complex BMC Biol 2010 8 29 10.1186/1741-7007-8-29 20359332 PMC2907588

[B47] Chaves LD Krueth SB Reed KM Characterization of the turkey MHC chromosome through genetic and physical mapping Cytogenet Genome Res 2007 117 213 220 10.1159/000103182 17675862

[B48] Nasar F Jankowski C Nag DK Long palindromic sequences induce double-strand breaks during meiosis in yeast Mol Cell Biol 2000 20 3449 3458 10.1128/MCB.20.10.3449-3458.2000 10779335 PMC85638

[B49] Nishant KT Rao MR Molecular features of meiotic recombination hot spots Bioessays 2006 28 45 56 10.1002/bies.20349 16369948

[B50] Itoh Y Kampf K Pigozzi MI Arnold AP Molecular cloning and characterization of the germline-restricted chromosome sequence in the zebra finch Chromosoma 2009 118 527 536 10.1007/s00412-009-0216-6 19452161 PMC2701497

[B51] Gordon L Yang S Tran-Gyamfi M Baggott D Christensen M Hamilton A Crooijmans R Groenen M Lucas S Ovcharenko I Stubbs L Comparative analysis of chicken chromosome 28 provides new clues to the evolutionary fragility of gene-rich vertebrate regions Genome Res 2007 17 1603 1613 10.1101/gr.6775107 17921355 PMC2045143

[B52] Muller C Denis M Gentzbittel L Faraut T The Iccare web server: an attempt to merge sequence and mapping information for plant and animal species Nucleic Acids Res 2004 32 W429 434 10.1093/nar/gkh460 15215424 PMC441598

[B53] Morisson M Lemière A Bosc S Galan M Plisson-Petit F Pinton P Delcros C Feve K Pitel F Fillon V Yerle M Vignal A ChickRH6: a chicken whole-genome radiation hybrid panel Genet Sel Evol 2002 34 521 533 10.1186/1297-9686-34-4-521 12270108 PMC2705459

[B54] de Givry S Bouchez M Chabrier P Milan D Schiex T CARHTA GENE: multipopulation integrated genetic and radiation hybrid mapping Bioinformatics 2005 21 1703 1704 10.1093/bioinformatics/bti222 15598829

[B55] Voorrips RE MapChart: software for the graphical presentation of linkage maps and QTLs J Hered 2002 93 77 78 10.1093/jhered/93.1.77 12011185

[B56] Beier DR Single-strand conformation polymorphism (SSCP) analysis as a tool for genetic mapping Mamm Genome 1993 4 627 631 10.1007/BF00360898 8281011

[B57] Budowle B Chakraborty R Giusti AM Eisenberg AJ Allen RC Analysis of the VNTR locus D1S80 by the PCR followed by high-resolution PAGE Am J Hum Genet 1991 48 137 144 1670750 PMC1682756

[B58] Crittenden LB Provencher L Santangelo L Levin I Abplanalp H Briles RW Briles WE Dodgson JB Characterization of a Red Jungle Fowl by White Leghorn Backcross Reference Population for Molecular Mapping of the Chicken Genome Poult Sci 1993 72 334 348

[B59] Bumstead N Palyga J A preliminary linkage map of the chicken genome Genomics 1992 13 690 697 10.1016/0888-7543(92)90143-G 1353476

[B60] Green P Falls K Crooks S Documentation for CRI-MAP, version 2.4 (3/26/90) Washington University School of Medicine, St Louis, MO 1990

