# Supplementary figures and images for: Integrative mapping analysis of chicken microchromosome 16 organization
Source: BMC Genomics. 2010 Nov 4;11:616. doi: 10.1186/1471-2164-11-616 (PMC3091757; doi:10.1186/1471-2164-11-616)

## Slide 1
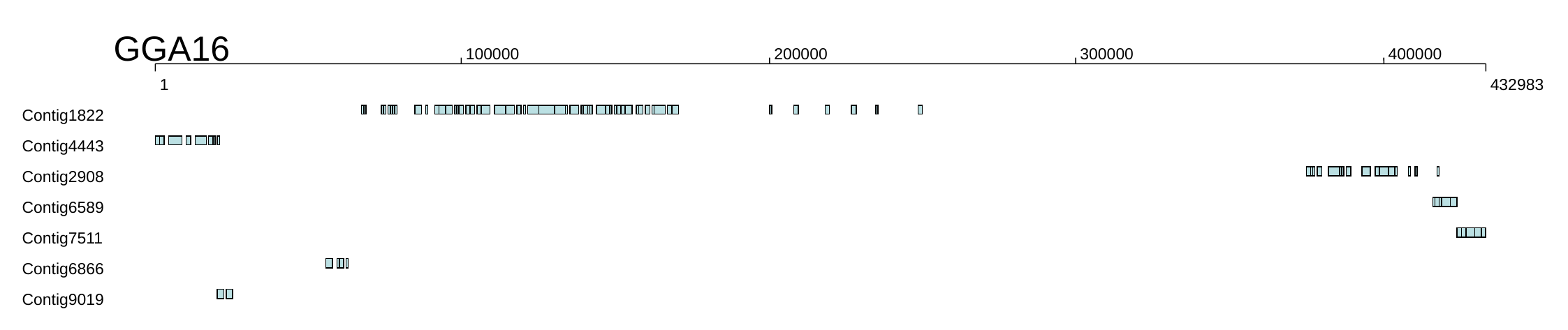

GGA16
 100000
 200000
 300000
 400000
 1
 432983
Contig1822
Contig4443
Contig2908
Contig6589
Contig7511
Contig6866
Contig9019

Supplement: Additional File 2 — 454 contigs distribution on the chicken sequence assembly. The 454 contigs (http://genome.wustl.edu/genomes/view/gallus_gallus/) were aligned to the available chicken sequence assembly (galGal 3, http://genome.ucsc.edu/index.html). The 7 matching contigs are represented along the GGA16 sequence. File format: PowerPoint [file 1471-2164-11-616-S2.PPT]
